# Changes in Rice Grain Quality of Indica and Japonica Type Varieties Released in China from 2000 to 2014

**DOI:** 10.3389/fpls.2017.01863

**Published:** 2017-10-31

**Authors:** Fan Feng, Yajun Li, Xiaoliang Qin, Yuncheng Liao, Kadambot H. M. Siddique

**Affiliations:** ^1^College of Agronomy, Northwest A&F University, Yangling, China; ^2^The UWA Institute of Agriculture, School of Agriculture and Environment, The University of Western Australia, Perth, WA, Australia

**Keywords:** inbred rice, hybrid rice, rice quality, japonica, indica, China

## Abstract

China is the first country to use heterosis successfully for commercial rice production. This study compared the main quality characteristics (head rice rate, chalky rice rate, chalkiness degree, gel consistency, amylose content, and length-to-width ratio) of 635 rice varieties (not including upland and glutinous rice) released from 2000 to 2014 to establish the quality status and offer suggestions for future rice breeding for grain quality in China. In the past 15 years, grain quality in japonica rice and indica hybrid rice has improved. In japonica rice, inbred varieties have increased head rice rates and decreased chalkiness degree over time, while hybrid rice varieties have decreased chalky rice rates and chalkiness degree. In indica hybrid rice, the chalkiness degree and amylose contents have decreased and gel consistency has increased. Improvements in grain quality in indica inbred rice have been limited, with some increases in head rice rate and decreases in chalky rice rate and amylose content. From 2010 to 2014, the percentage of indica varieties meeting the Grade III national standard of rice quality for different quality traits was low, especially for chalky rice rate and chalkiness degree. Japonica varieties have more superior grain quality than indica rice in terms of higher head rice rates and gel consistency, lower chalky rice rates and chalkiness degree, and lower amylose contents, which may explain why the Chinese prefer japonica rice. The japonica rice varieties, both hybrid and inbred, had similar grain qualities, but this varied in indica rice with the hybrid varieties having higher grain quality than inbred varieties due to significantly better head rice rates and lower chalkiness degree. For better quality rice in future, the chalky rice rate and chalkiness degree should be improved in japonica rice along with most of the quality traits in indica rice.

## Introduction

Rice (*Oryza sativa*) is the staple food for approximately 65% of the Chinese population (Xin and Li, 2009). Historically, breeding programs in China primarily aimed at improving rice yield potential, which increased rice yields from 1890 kg ha^-1^ in 1949 to 6662 kg ha^-1^ in 2013 (Wang et al., 2015). The average rice yield in China is almost 50% higher than the worldwide average (Zhao, 2008; Liu and Ren, 2014). The world population is expected to reach 9 billion people by 2050^1^. Grain production needs to increase by approximately 44 million metric tons per year to ensure sufficient food production to feed this population (Tester and Langridge, 2010). China imported 3.56 million tons of rice in National Bureau of Statistics of China (2016) ^2^. Meanwhile, in China, with improvements in economic and living standards, the grain quality of rice has become the most important factor for rice production as it is directly related to its market value, and thus influences farmer income (Yang et al., 2004; Zhang, 2007).

Rice quality traits include milling recovery, physical appearance, cooking and eating characteristics, and nutritional value (Juliano, 1972, 1985; Perez and Juliano, 1978; Mo, 1993; Lisle et al., 2000; Bao et al., 2002; Han et al., 2004; Koutroubas et al., 2004; Cheng et al., 2005). In the United States and other countries, the evaluation of rice quality includes the viscosity of rice noodles [Rapid Visco Analyzer (RVA) spectrum] and the texture of steamed rice (Ryu et al., 1993; Zhang et al., 2008). It has long been accepted that while the yield of hybrid rice is high, its quality is poor in China (Lu et al., 2001; Min et al., 2007; Zhao, 2008). To improve rice breeding for grain quality, the Ministry of Agriculture of China promulgated the agricultural industry standards: NY20-1986 (1986) ‘quality edible rice,’ GB/T17891-1999 (1999) ‘quality rice’ (Supplementary Table S1) and NY/T-593-2002 (2002) ‘cooking rice variety quality.’ Many studies have shown that rice quality is mainly controlled by genes (Tan et al., 1999; Mikami, 2000; Tran et al., 2011), so breeders in China aimed to breed high-quality rice varieties (Yang et al., 2004).

Rice can be divided into hybrid or inbred varieties within japonica (*Oryza sativa* L. subsp. *japonica*) and indica (*Oryza sativa* L. subsp. *indica*) (Catudan and Arocena, 2003; Tripp et al., 2010; Min et al., 2012). The successful development of hybrid rice, which significantly increased rice yield, is noteworthy in the history of modern agriculture (Virmani, 1994; Zhao, 2008; Horgan and Crisol, 2013) with China being the first country to use heterosis successfully for the commercial production of rice (Wang, 2004). In China, indica rice is planted mainly in southern China (Cheng, 1993; Qian, 2007; Min et al., 2011, 2012) with mostly hybrid varieties (Deng et al., 2006). Japonica rice is planted mainly in the northeast plain and Yangtze River region (Cheng, 1993; Qian, 2007; Wang et al., 2015) and is dominated by inbred varieties (Deng et al., 2006). There has been none research on the improvement of grain quality over time for the different types of rice in China.

In this study, the main quality characteristics of 635 rice varieties were compared to establish their quality status and to identify strategies for future grain quality improvements in rice in China.

## Materials and Methods

### Data Collection

Data related to the main quality characteristics of 635 rice varieties (not including upland and glutinous rice)—all certified by the national or provincial crop variety appraisal committees from 2000 to 2014—were collated from published reports (Supplementary Data Sheet 1). The collated data included head rice rate (the percentage of the total weight that is head rice), chalky rice rate (the percentage of total grains that is chalky), chalkiness degree (the percentage of the total area of a kernel), gel consistency (the flow length of rice gel after rice flour pasting), amylose content and the length-to-width ratio (see Supplementary Presentation 1 and Wang et al., 2011). Data for the length-to-width ratio is included for indica rice but not japonica rice because there is no requirement in the GB /T17891-1999 high-quality rice standard to evaluate length-to-width ratio in japonica rice. The rice varieties were divided into four types: japonica inbred (119 varieties), japonica hybrid (64), indica inbred (24), and indica hybrid (428). The two main planting regions for indica rice are the Yangtze River region (annual rainfall 1000–2000 mm, sunshine hours 1100–2500 h, cumulative total temperatures above 10°C (CT) 4500–5800°C where CT = {mean daily temperature – 10}× number of days, and main soil types are red and paddy soils) and the south coast region (annual rainfall 1100–3000 mm, sunshine hours 1300–2600 h, CT 5000–9300°C, and main soil type is red soil), both of which have abundant light, temperature and water resources. Japonica rice is mainly planted in the northeast plain (annual rainfall 320–1000 mm, sunshine hours 2400–3100 h, CT 2000–3600°C, and main soil type is black soil) and in the Yangtze River region (detailed above).

### Data Analyses

Statistical Analysis System (SAS) software was used to compare the quality characteristics between rice varieties; least significant difference (LSD) values were calculated at the 5% probability level. The effects of subspecies (japonica and indica rice), breeding type (hybrid, inbred), year of release, and their interactions on grain quality traits in rice were performed using PROC MIXED in the SAS software (SAS Institute, 2000). Regression analysis with a standard linear model applied to variety means was used to calculate the relative (%) genetic gains of each grain quality trait with year of release.

$$\begin{array}{c} \mathrm{yi}\;=\;a+\mathrm{bxi}+u \end{array}$$

where yi is the value for grain quality, xi is the year in which cultivar i was released, a is the intercept of both equations, b is the absolute slope, and u is the residual error.

According to their year of release, the varieties were divided into three groups: 2000–2004, 2005–2009, and 2010–2014. The percentage of each variety type meeting the national standards for each quality characteristic were calculated according to the Ministry of Agriculture standard GB/T17891-1999 ‘high quality rice.’

## Results

### Effects of Subspecies, Breeding Type, Year of Release, and Their Interactions on Yield and Grain Quality Traits in Rice

Most of the assessed traits were significantly affected by subspecies, breeding type and year of release. Significant subspecies × breeding type interactions were observed for head rice rate and chalkiness degree. Significant subspecies × year of release interactions were observed for head rice rate, chalky rice rate and gel consistency. Significant breeding type × year of release interactions were observed for gel consistency, amylose content and length-to-width ratio. Significant subspecies × breeding type × year of release interactions were observed for chalky rice rate, chalkiness degree and amylose content (**Table 1**).

**Table 1:** The *F*-values for the effects of subspecies, breeding type, year of release and their interaction on grain quality traits of rice.

Factors	Head rice rate	Chalky rice rate	Chalkiness degree	Gel consistency	Amylose content	Length-to-width ratio
Subspecies (japonica, indica)	50.38***	33.60***	2.83ns	245.68***	142.38***	–
Breeding type (hybrid, inbred)	579.91***	82.83***	39.13***	639.78***	629.30***	0.20ns
Year of release	54.76***	17.23***	23.04***	86.48***	5.71**	5.37**
Subspecies × breeding type	9.81**	0^ns^	40.33***	0^ns^	0^ns^	–
Subspecies × year of release	27.17***	11.23***	0.73ns	37.34***	2.76ns	–
Breeding type × year of release	0^ns^	0^ns^	0^ns^	40.95***	5.3**	53.72***
Subspecies × breeding type × year of release	0^ns^	31.93***	8.12***	0^ns^	22.43***	–

*^∗∗^Significant at 0.01 probability level; ^∗∗∗^Significant at 0.001 probability level; ns, not significant.*

### Comparison of Quality Traits between Rice Types

Japonica rice had significantly higher head rice rates and yields than indica rice (*P* < 0.05). For indica rice, hybrid varieties had significantly higher head rice rates than inbred varieties, but there was no difference between japonica inbred and japonica hybrid rice. Indica rice had significantly higher chalkiness degree and chalky rice rates than japonica rice (*P* < 0.05). Indica inbred rice had significantly higher chalkiness degree than indica hybrid rice, but there was no difference for chalky rice rate. Chalkiness degree and chalky rice rates did not differ between japonica inbred and japonica hybrid rice. Japonica rice had significantly lower amylose contents than indica rice (*P* < 0.05), but no difference between hybrid and inbred rice for either rice type. For indica rice, the length-to-width ratio did not differ between hybrid and inbred rice (**Table 2**).

**Table 2:** The difference in mean quality traits between japonica inbred rice, japonica hybrid rice, indica inbred rice, and indica hybrid rice.

Rice type	*n*	Head rice rate (%)	Chalky rice rate (%)	Chalkiness degree (%)	Gel consistency (mm)	Amylose content (%)	Length-to-width ratio
Japonica inbred	119	67.06a	21.92c	3.13c	81.15a	16.84b	–
Japonica hybrid	64	67.86a	29.01c	4.76c	78.36a	16.27b	–
Indica inbred	24	52.82c	45.46a	12.19a	65.82c	20.29a	2.87a
Indica hybrid	428	58.38b	41.00a	7.40b	64.08c	20.37a	2.88a

*Mean values followed by the same letter are not significantly different at the 5% probability level.*

### Genetic Gain for Different Quality Traits From 2000 to 2014

The grain quality of japonica rice increased from 2000 to 2014; for inbred rice, the head rice rate increased and chalkiness degree decreased, and for hybrid rice, the chalky rice rate and chalkiness degree decreased, with no changes in the other quality traits (**Table 3**). The changes in grain quality from 2000 to 2014 of indica rice varied between hybrid and inbred varieties. For indica hybrid rice, grain quality increased with decreased chalkiness degree and amylose content and increased gel consistency. For indica inbred rice, the head rice rate increased significantly, suggesting an increase in grain quality, but the chalky rice rate and amylose content increased and length-to-width ratio decreased, which ultimately reduced grain quality (**Table 3**).

**Table 3:** Genetic gain (%) in head rice rate, chalky rice rate, chalkiness degree, gel consistency, amylose content and length-to-width ratio in inbred and hybrid rice varieties registered from 2000 to 2014.

Rice type	Head rice rate	Chalky rice rate	Chalkiness degree	Gel consistency	Amylose content	Length-to-width ratio
Japonica inbred	0.38^∗∗^	ns	–5.74^∗∗^	ns	ns	–
Japonica hybrid	ns	–4.24^∗^	–7.09^∗∗^	ns	ns	–
Indica inbred	4.49^∗∗^	70.83^∗∗^	ns	ns	3.36^∗^	–3.00^∗∗^
Indica hybrid	ns	ns	–1.95^∗^	3.02^∗∗^	–0.89^∗∗^	ns

*^∗^Significant at 0.05 probability level; ^∗∗^Significant at 0.01 probability level; ns, not significant.*

### Variation Coefficient for Different Quality Traits in Inbred and Hybrid Rice

For the three periods of rice cultivation (2000–2004, 2005–2009, and 2010–2014), chalky rice rate and chalkiness degree had the highest variation coefficients, while the other four quality indices had relatively low variation coefficients. Breeding reduced the variation coefficient for chalky rice rate and chalkiness degree in japonica and indica inbred varieties. Indica hybrid rice had lower variation coefficients for gel consistency than indica inbred rice, which decreased over time. Variation coefficients for amylose content in japonica and indica rice also decreased over time (**Table 4**).

**Table 4:** The variation coefficients for quality traits of inbred and hybrid rice varieties registered from 2000 to 2014.

Rice type	Year	Head rice rate	Chalky rice rate	Chalkiness degree	Gel consistency	Amylose content	Length-to-width ratio
Japonica inbred	2000–2004	7.37	76.8	88.58	7.81	6.44	–
	2005–2009	**4.95**	61.18	63.67	5.06	5.11	–
	2010–2014	**4.97**	**47.6**	**58.81**	**3.65**	**4.57**	–
	Mean	5.76ab	61.86a	70.35a	5.51c	5.37a	–
Japonica hybrid	2000–2004	5.89	44.54	65.25	11.22	8.87	–
	2005–2009	6.25	44.37	54.28	7.55	16.24	–
	2010–2014	**4.23**	57.43	76.72	**7.04**	**3.69**	–
	Mean	5.46b	48.78a	65.42a	8.6c	9.6a	–
Indica inbred	2000–2004	14.66	114.91	165.68	23.24	27.06	8.39
	2005–2009	15.71	70.07	76.5	23.32	26.81	18.35
	2010–2014	**4.24**	**3.72**	**22.19**	26.51	**4.21**	**7.51**
	Mean	11.54ab	62.9a	88.12a	24.36	19.36a	11.42a
Indica hybrid	2000–2004	13.15	53.66	79.62	22.74a	11.68	11.34
	2005–2009	12.56	65.24	78.68	**17.08**	16.07	10.73
	2010–2014	**10.45**	57.44	**70.38**	**18.03**	18.81	**9.03**
	Mean	12.05a	58.78a	76.23a	19.28b	15.52a	10.37a

*Bold values mean that variation of traits was lower when compared with those of older varieties; mean values followed by the same letter do not significantly differ at the 5% probability level.*

### Percentage of Each Variety Type Meeting the Grade III National Standard for Rice Quality Traits

For head rice rate, hybrid rice had a higher percentage of varieties that met the Grade III national standard of rice quality (GB/T17891-1999, China) than inbred rice from 2000 to 2014, japonica rice had a higher percentage of varieties that met the Grade III national standard than indica rice in most periods. For chalky rice rate, japonica rice had a higher percentage of rice varieties that met the Grade III national standard for chalky rice rate than indica rice; a low and highly variable (0–70%) percentage of indica inbred and hybrid rice varieties met the standard. Japonica rice had a much higher percentage of varieties that met the Grade III national standard for chalkiness degree, amylose content and gel consistency than indica rice in most periods; from 2000 to 2014, japonica inbred rice had a higher percentage for chalkiness degree and similar percentage for amylose content and gel consistency than japonica hybrid rice. Indica hybrid rice had higher mean percentage for gel consistency than indica inbred rice, and higher chalkiness degree, gel consistency, amylose content, and length-to-width ratio than indica inbred rice in most time periods (**Table 5**).

**Table 5:** The percentage of each variety meeting Grade III quality standards for different quality traits.

Rice type	Year	Number of varieties	Head rice rate	Chalky rice rate	Chalkiness degree	Gel consistency	Amylose content	Length-to-width ratio
Japonica inbred	2000–2004	35	74.3	71.9	60.0	85.7	97.1	–
	2005–2009	49	93.9	79.6	100.0	100.0	98.0	–
	2010–2014	35	94.3	71.4	94.3	100.0	100.0	–
	2000–2014		87.5	74.3	84.8	95.2	98.4	–
Japonica hybrid	2000–2004	17	88.2	33.3	29.4	93.3	94.1	–
	2005–2009	36	100.0	86.1	83.3	100.0	94.4	–
	2010–2014	11	100.0	63.6	63.6	100.0	90.9	–
	2000–2014		96.1	61.0	58.8	97.8	93.2	–
Indica inbred	2000–2004	13	38.5	70.0	38.5	81.8	46.2	88.9
	2005–2009	8	62.5	37.5	37.5	75.0	25.0	62.5
	2010–2014	3	100.0	0.0	0.0	66.7	0.0	0.0
	2000–2014		67.0	35.8	25.3	74.5	23.7	50.5
Indica hybrid	2000–2004	93	77.4	33.3	37.4	52.7	85.0	95.0
	2005–2009	174	87.4	50.6	54.6	90.2	85.6	74.7
	2010–2014	161	84.5	45.3	47.8	91.9	78.3	71.4
	2000–2014		83.1	43.1	46.6	78.3	83.0	80.4

## Discussion

### Change in Different Grain Quality with Breeding Selection

Milling quality in rice refers to its characteristics after milling (Lisle et al., 2000). The head rice rate is a major limiting factor for processing quality and market value in rice (Liao et al., 2003). In this study, the head rice rate did not significantly differ between japonica inbred rice and japonica hybrid rice. The percentage of japonica rice varieties that meet the Grade III national standard for head rice rate reached at least 93% in two periods (2005–2009, 2010–2014), which is consistent with the results of Zhao (2008). Indica rice had significantly lower head rice rates than japonica rice from 2000 to 2014, which is consistent with the results of Min et al. (2008). The head rice rate in indica inbred rice increased from 2000 to 2014, but there was no change for indica hybrid rice. The mean percentage of indica rice varieties that met the Grade III national standards for head rice rate from 2010 to 2014 was <85%, lower than japonica rice (≥94%). This is an important quality trait that needs to be improved in indica rice.

Appearance quality, also known as commodity quality, mainly includes chalky characters, chalky area and the length-to-width ratio (Lisle et al., 2000). High chalky rice rates and chalkiness degree have become major obstacles to improving rice grain quality in China (Xu et al., 1995, 2003; Du et al., 2007). In this study, japonica rice had a lower chalky rice rate and chalkiness degree than indica rice, which is likely to decrease further according to the trends from 2000 to 2014. From 2000 to 2014, the chalky rice rate increased in indica inbred rice, but neither the chalky rice rate nor chalkiness degree changed in indica hybrid rice. However, the percentage of indica rice varieties that met the Grade III national standards for chalky rice rate and chalkiness degree from 2010 to 2014 was very low. While the chalky rice rate and chalkiness degree have a small impact on rice taste, they are critical appearance quality traits in the domestic rice market and for foreign trade (Jin, 2011). It is important and urgent to improve these appearance quality traits in indica rice grains.

From 2000 to 2014, the length-to-width ratio decreased in indica inbred rice, but there was no change in indica hybrid rice. However, the percentage of indica inbred rice varieties that met the national standards of quality rice for length-to-width ratio was low at 50.5% compared with 80.4% in indica hybrid rice. Greater emphasis on improving this characteristic in indica rice is needed in future breeding programs.

Cooking and eating qualities mainly refer to characteristics in the cooking process, which include gel consistency and amylose content (Juliano, 1972; Perez and Juliano, 1978; Bao et al., 2002). Higher gel consistency means softer and better quality rice. In our study, japonica rice had significantly higher gel consistency than indica rice, which agrees with the results of Zhu et al. (2004), who collated data from 1985 to 2002. The gel consistency in japonica rice did not change from 2000 to 2014, but more than 95% of the varieties met the Grade III national standard (GB/T17891-1999, China) for gel consistency. From 2000 to 2014, gel consistency increased in indica hybrid rice and did not change in indica inbred rice. Only 74.50 and 78.28% of the indica inbred and indica hybrid varieties met the Grade III national standard (GB/T17891-1999, China) for gel consistency, which suggests that there is room for improvement in both indica inbred and indica hybrid rice.

The amylose content in rice affects the softness and palatability of steamed rice and is closely correlated with the hardness, cohesiveness, and viscosity of rice. If the amylose content is too high, steamed rice will have poor viscosity and rice extension, high pasting temperature, and low palatability. If the amylose content is low, steamed rice will be soft with better viscosity and gloss, but if it is too low, the viscosity will be too high, making the steamed rice taste greasy and unsuitable for boiling. From 2000 to 2014, amylose content did not change in japonica inbred rice, but it declined in japonica hybrid rice. The percentage of japonica varieties that met the Grade III national standard for amylose content was >90%. The amylose content declined in indica hybrid rice but increased in indica inbred rice. The percentage of indica varieties that met the Grade III national standard was much lower than japonica rice, owing to their high amylose contents.

### Difference in Grain Quality between Inbred and Hybrid, Indica and Japonica Rice

At present, China has the larger planting areas of indica hybrid and japonica inbred rice than indica inbred and japonica hybrid rice. Japonica inbred rice has better quality grain than indica hybrid rice based on all grain quality traits in this study, which is consistent with the findings of Zhu et al. (2004), who compared data on japonica and indica rice collated from 1985 to 2002 in China. The quality of hybrid rice was poor due to crosses with indica rice varieties that have high chalky rice rates and chalkiness degree, which affect appearance quality, and low gel consistencies and high amylose contents, which affect cooking and eating qualities. The Chinese prefer japonica inbred rice over indica hybrid rice, which explains why many there is a perception that hybrid rice has poor quality. Our study found that japonica hybrid rice has similar grain quality to japonica inbred rice, which differs from the findings of an earlier study (Yang et al., 2004). Indeed, indica hybrid rice had better quality grain than indica inbred rice, with its significantly higher head rice rate and lower chalkiness degree, and no differences in the other grain quality traits (**Table 2**), which again differs from the findings of Yang et al. (2004).

### Challenge of Grain Quality under Climate Change

Japonica rice is popular in China because of its good grain quality and high yield. Indica rice is mainly grown in southern China, due to suitable climatic conditions in the region. Indica rice generally produces higher yields and has shorter growth duration (Lu et al., 2001; Min et al., 2007; Zhao, 2008). However, the poor grain quality or indica rice limits its use in cooking. Temperature and atmospheric CO_2_ concentration are important factors affecting the formation of rice grain quality (Wang et al., 2011). Atmospheric CO_2_ concentrations have increased and global temperatures are rising (Schönwiese et al., 1995; NOAA, 2013). Studies have shown that high CO_2_ concentrations and temperatures can worsen rice grain quality of milling recovery and physical appearance (Wang et al., 2011; Madan et al., 2012; Usui et al., 2014). Temperatures is higher in southern than north China, increaseing trends of global temperatures are not conducive to improving grain quality, especially for indica rice in southern China with high temperature. Future research should focus on adapting rice to the effects of increased CO_2_ concentrations and temperatures to improve grain quality and yield.

## Conclusion

China is a major rice-producing country and is acutely aware of the importance of improving the quality and yield of rice. In the past 15 years, grain quality in japonica rice and indica hybrid rice have improved, while there have been limited improvements in indica inbred rice—the head rice rate has increased, but the chalky rice rate and amylose content have decreased. The percentage of indica varieties meeting the Grade III national standard of rice quality for different quality traits is low, especially for chalky rice rate and chalkiness degree.

In japonica rice, the chalky rice rate and chalkiness degree could be improved for better grain quality. In indica rice, new germplasm resources need to be identified, and the introgression of relevant gene/s from japonica may improve the quality traits.

## Author Contributions

Contributed the experimental design: XQ and YcL. Data collection: FF, YL, and XQ. Analyzed the data: XQ, FF, and YL. Wrote the paper: FF, YL, KS, YcL, and XQ. All authors read and approved the final manuscript.

## Conflict of Interest Statement

The authors declare that the research was conducted in the absence of any commercial or financial relationships that could be construed as a potential conflict of interest.

## Footnotes

1 http://www.fao.org/wsfs/world-summit/en/

2 www.stats.gov.cn

## References

[B1] Bao J. S. Sun M. Corke H. (2002). Analysis of the genetic behavior of some starch properties in indica rice (*Oryza sativa* L.): thermal properties, gel texture, swelling volume. *Theor. Appl. Genet.* 104 408–413. 10.1007/s001220100688 12582713

[B2] Catudan B. M. Arocena A. C. (2003). Determinants of the adoption of F1 hybrid rice in the Philippines. *Philos. Rice Tech. Bull.* 8 64–69.

[B3] Cheng F. M. Zhong L. J. Wang F. Zhang G. P. (2005). Differences in cooking and eating properties between chalky and translucent parts in rice grains. *Food Chem.* 90 39–46. 10.1016/j.foodchem.2004.03.018

[B4] Cheng K. S. (1993). *The Identification of O. sativa L. subsp. Ting and O. sativa L. subsp. Keng Ting.* Kunming: Yunnan Science and Technology Press.

[B5] Deng H. F. He Q. Shu F. (2006). Research status and countermeasures of japonica hybrid rice in China. *Hybrid Rice* 21 1–6.

[B6] Du Y. Wang Y. Wang X. H. Sun N. Yang J. C. (2007). Comparisons of plant type, grain yield, and quality of different japonica rice cultivars in Huanghe-Huaihe River Area. *Acta Argron. Sin.* 33 1079–1085.

[B7] GB/T17891-1999 (1999). *High Quality Paddy.* Beijing: Standardization Administration of the People’s Republic of China.

[B8] Han Y. Xu M. Liu M. Yan C. Korban S. S. Chen X. (2004). Genes coding for starch branching enzymes are major contributors to starch viscosity characteristics in waxy rice (*Oryza sativa* L.). *Plant Sci.* 166 357–364. 10.1016/j.plantsci.2003.09.023

[B9] Horgan F. G. Crisol E. (2013). Hybrid rice and insect herbivores in Asia. *Entomol. Exp. Appl.* 148 1–19. 10.1111/eea.12080 26072143

[B10] Jin H. M. (2011). Comparison of main traits of indica hybrid rice and inbred conventional rice in middle and lower stream of the Yangtze River. *China Rice* 17 14–16.

[B11] Juliano B. O. (1972). “The rice caryopsis and its composition,” in *Rice Chemistry and Technology* ed. Houston D. F. (Saint Paul MN: AACC Publications).

[B12] Juliano B. O. (1985). “Rice: chemistry and technology,” in *The American Association of Cereal Chemists* Vol. 2 ed. Juliano B. O. (Saint Paul MN: AACC Publications) 443–524. 10.1080/0142968X.1985.11904307

[B13] Koutroubas S. D. Mazzini F. Pons B. Ntanos D. A. (2004). Grain quality variation and relationships with morpho-physiological traits in rice (*Oryza sativa* L.) genetic resources in Europe. *Field Crop Res.* 86 115–130. 10.1016/S0378-4290(03)00117-5

[B14] Liao F. M. Zhou K. L. Yang H. H. Xu Q. S. (2003). Comparison of grain quality between f1 hybrids and their parents in indica hybrid rice. *Chin. J. Rice Sci.* 17 134–140.

[B15] Lisle A. J. Martin M. Fitzgerald M. A. (2000). Chalky and translucent rice grains differ in starch composition and structure and cooking properties. *Cereal Chem.* 77 627–632. 10.1016/S0378-4290(03)00117-5

[B16] Liu X. R. Ren H. X. (2014). Analysis of Chinese rice market in 2013 and prospect in 2014. *North Rice* 44 1–5.

[B17] Lu Q. S. Hua Z. T. Zou J. C. (2001). *Heterosis of Crops.* Beijing: China Agricultural Press.

[B18] Madan P. Jagadish S. V. K. Craufurd P. Q. Fitzgerald M. Lafarge T. Wheeler T. R. (2012). Effect of elevated CO2 and high temperature on seed-set and grain quality of rice. *J. Exp. Bot.* 63 3843–3852. 10.1093/jxb/ers077 22438302 PMC3388820

[B19] Mikami I. (2000). Effects of the two common Wx alleles on different genetic backgrounds in rice. *Plant Breed.* 119 505–508. 10.1046/j.1439-0523.2000.00533.x

[B20] Min J. Zhu Z. W. Chen N. (2012). Study on the quality and the percentages meeting fine quality standards in Chinese indica conventional rice. *China Rice* 18 4–7.

[B21] Min J. Zhu Z. W. Jin L. D. Xu L. Zhang L. P. Tang S. X. (2011). Analysis on grain quality of indica hybrid rice combinations bred during twenty-five years in China. *Chin. J. Rice Sci.* 25 201–205.

[B22] Min J. Zhu Z. W. Xu L. (2008). Analysis of grain quality and superior quality rate of glutinous rice cultivars bred since the 1980s of the 20th century in China. *China Rice* 43 1–4.

[B23] Min J. Zhu Z. W. Xu L. Mou R. (2007). Studies on grain quality and high quality of japonica hybrid rice in China. *Hybrid Rice* 22 67–70. 17704538

[B24] Mo H. D. (1993). Improvement of rice quality in China. *Chin. Agr. Sci.* 26 8–14.

[B25] National Bureau of Statistics of China (2016). *China Statistical Yearbook.* Beijing: China Statistics Press.

[B26] NOAA (2013). Available at: http://www.esrl.noaa.gov/gmd/ccgg/trends/ (accessed January 27 2014).

[B27] NY/T-593-2002 (2002). *Cooking Rice Variety Quality.* Beijing: Standardization Administration of the People’s Republic of China.

[B28] NY20-1986 (1986). *High Quality Edible Rice.* Beijing: Standardization Administration of the People’s Republic of China.

[B29] Perez C. M. Juliano B. O. (1978). Modification of the simplified amylose test for milled rice. *Starch Starke* 30 424–426. 10.1002/star.19780301206

[B30] Qian Q. (2007). *Breeding by Gene Design in Rice* Beijing: Science Press.

[B31] Ryu G. H. Neumann P. E. Walker C. E. (1993). Pasting of wheat flour extrudates containing conventional baking ingredients. *J. Food Sci.* 58 567–573. 10.1111/j.1365-2621.1993.tb04325.x

[B32] SAS Institute (2000). *SAS user’s Guide: Statistics.* Cary, NC: SAS Inst.

[B33] Schönwiese C. Houghton J. T. Meira Filho L. G. Callander B. A. Harris N. Kattenberg A. (eds) (1995). *Climate Change The Science of Climate Change.* Cambridge: Cambridge University Press.

[B34] Tan Y. F. Li J. X. Yu S. B. Xing Y. Z. Xu C. G. Zhang Q. F. (1999). The three important traits for cooking and eating quality of rice grains are controlled by a single locus in an elite rice hybrid, Shanyou 63. *Theor. Appl. Genet.* 99 642–648. 10.1007/s001220051279 22665200

[B35] Tester M. Langridge P. (2010). Breeding technologies to increase crop production in a changing world. *Science* 327 818–822. 10.1126/science.1183700 20150489

[B36] Tran N. A. Daygon V. D. Resurreccion A. P. Cuevas R. P. Corpuz H. M. Fitzgerald M. A. (2011). A single nucleotide polymorphism in the Waxy gene explains a significant component of gel consistency. *Theor. Appl. Genet.* 123 519–525. 10.1007/s00122-011-1604-x 21562821

[B37] Tripp R. Hu R. Pal S. (2010). “Rice seed provision and the evolution of seed markets,” in *Rice in the Global Economy: Strategic Research and Policy Issues for Food Security* eds Pandey S. Byerlee D. Dawe D. Dobermann A. Mohanty S. Rozelle S. (Los Baños: IRRI).

[B38] Usui Y. Sakai H. Tokida T. Nakamura H. Nakagawa H. Hasegawa T. (2014). Heat-tolerant rice cultivars retain grain appearance quality under free-air CO2 enrichment. *Rice* 7 6–9. 10.1186/s12284-014-0006-5 24920972 PMC4052674

[B39] Virmani S. S. (1994). “Prospects of hybrid rice in the tropics and the subtropics,” in *Hybrid Rice Technology: New Developments and Future Prospects* ed. Virmani S. S. (Los Baños: IRRI).

[B40] Wang C. L. (2004). Status and prospects of hybrid rice breeding in Jiangsu. *China Rice Sci.* 12 219–225.

[B41] Wang Y. X. Frei M. Song Q. L. Yang L. X. (2011). The impact of atmospheric CO2 concentration enrichment on rice quality—A research review. *Acta Ecol. Sin.* 31 277–282. 10.1016/j.chnaes.2011.09.006

[B42] Wang Y. Z. Wang X. J. Li Y. Xu H. Wang J. Y. Zhao M. H. (2015). Analysis of yield and quality traits and their relationship in japonica rice in northern China. *Acta Agron. Sin.* 41 84–92. 10.3724/SP.J.1006.2015.00910

[B43] Xin L. J. Li X. B. (2009). Changes of multiple cropping in double cropping rice area of southern China and its policy implications. *J. Natl. Res.* 24 58–65.

[B44] Xu F. X. Zheng J. K. Zhu Y. C. Wang G. X. (2003). Effect of atmospheric phenomena factors on the milling quality and the appearance quality of medium india hybrid rice during the period from full heading to maturity. *Chin. J. Plant Ecol.* 27 73–77. 10.17521/cjpe.2003.0011

[B45] Xu Q. R. Chen K. R. Chang Y. K. Guan Y. Z. (1995). Current status of hybrid rice quality and its improving measures. *Hybrid Rice* 9 28–32.

[B46] Yang Y. S. Chen L. Y. Xu Y. W. (2004). See the development of quality breeding in China from the change of rice quality evaluation standard. *Hybrid Rice* 19 5–10.

[B47] Zhang Q. (2007). Strategies for developing green super rice. *Proc. Natl. Acad. Sci. U.S.A.* 104 16402–16409. 10.1073/pnas.0708013104 17923667 PMC2034246

[B48] Zhang Z. Zhang S. Yang J. Zhang J. (2008). Yield, grain quality and water use efficiency of rice under non-flooded mulching cultivation. *Field Crop Res.* 108 71–81. 10.1016/j.fcr.2008.03.004

[B49] Zhao J. (2008). Comparison of grain yield and quality between hybrid rice and conventional rice in China. *Hybrid Rice* 23 1–4.

[B50] Zhu Z. W. Chen N. Wang D. Y. Zhang X. F. Yao Q. Min J. (2004). Analysis on variation and difference for rice quality traits among different types of rice. *Chin. J. Rice Sci.* 18 315–320.

