# Supplementary material for: Changes in Rice Grain Quality of Indica and Japonica Type Varieties Released in China from 2000 to 2014
Source: Front Plant Sci. 2017 Oct 31;8:1863. doi: 10.3389/fpls.2017.01863 (PMC5671604; doi:10.3389/fpls.2017.01863)
Supplement: Supplementary file 1 [file Table_1.DOCX]

**S1 Supplementary TABLE S1** Classification of high-quality rice quality from GB/T17891-1999

| Rice type | Grade | Brown rice rate (%) | Head rice rate (%) | Chalky rice rate (%) | Chalkiness degree (%) | Amylose content (%) | Grade of eating quality | Gel consistency  (mm) | Length-to-width ratio | Imperfect rice (%) | Other varieties of rice (%) | Yellow grain rice (%) | Impurity (%) | Water content (%) | Color and luster |
| --- | --- | --- | --- | --- | --- | --- | --- | --- | --- | --- | --- | --- | --- | --- | --- |
| Indica | 1 | ≥79 | ≥56 | ≤10 | ≤1 | 17–22 | ≥9 | ≥70 | ≥2.8 | ≤2 | ≤1 | ≤0.5 | ≤1 | ≤13.5 | normal |
|  | 2 | ≥77 | ≥54 | ≤20 | ≤3 | 16–23 | ≥8 | ≥60 | ≥2.8 | ≤3 | ≤2 | ≤0.5 | ≤1 | ≤13.5 | normal |
|  | 3 | ≥75 | ≥52 | ≤30 | ≤5 | 15–24 | ≥7 | ≥50 | ≥2.8 | ≤5 | ≤3 | ≤0.5 | ≤1 | ≤13.5 | normal |
| Japonica | 1 | ≥81 | ≥66 | ≤10 | ≤1 | 15–18 | ≥9 | ≥80 | – | ≤2 | ≤1 | ≤0.5 | ≤1 | ≤14.5 | normal |
|  | 2 | ≥79 | ≥64 | ≤20 | ≤3 | 15–19 | ≥8 | ≥70 | – | ≤3 | ≤2 | ≤0.5 | ≤1 | ≤14.5 | normal |
|  | 3 | ≥77 | ≥62 | ≤30 | ≤5 | 15–20 | ≥7 | ≥60 | – | ≤5 | ≤3 | ≤0.5 | ≤1 | ≤14.5 | normal |
| Long-grain glutinous | – | ≥77 | ≥54 | – | – | ≤2.0 | ≥7 | ≥100 | – | ≤5 | ≤3 | ≤0.5 | ≤1 | ≤13.5 | normal |
| Japonica glutinous | – | ≥80 | ≥60 | – | – | ≤2.0 | ≥7 | ≥100 | – | ≤5.0 | ≤3 | ≤0.5 | ≤1 | ≤14.5 | normal |
