# Supplementary material for: Changes in Rice Grain Quality of Indica and Japonica Type Varieties Released in China from 2000 to 2014
Source: Front Plant Sci. 2017 Oct 31;8:1863. doi: 10.3389/fpls.2017.01863 (PMC5671604; doi:10.3389/fpls.2017.01863)
Supplement: Supplementary file 2 [file Presentation_1.PDF]

Method for test head rice rate

When the brown rice is milled to head rice, it was easily broken. Head rice refers to rice kernels with lengths greater than or equal to three-fourths of their normal length. Head rice rate is expressed as the percentage of the total weight that is head rice. The test method for head rice rate is as follows: the net weight of the rice samples was measured using a weighing balance. After shelling, the brown rice was weighed and milled using a milling machine (accuracy for the national level standards).The bran powder was removed, and the head rice picked and weighed. The head rice rate was calculated using formula (1):

$$H(\%) = \frac{H3}{H2} \times \frac{H1}{H0} \times 100 \quad (1)$$

where H is the head rice rate, H0 is the net weight of rice samples, H1 is the weight of shelled rice, H2 is the weight of brown rice, and H3 is the weight of head rice.

Method for test chalky rice rate

Chalky rice rate is expressed as the percentage of total grains that is chalky. The test method for chalky rice rate is as follows: chalkiness was evaluated visually on 100 milled grains per plot. Grains with ≥20% white belly, white center, white back, or a combination of these were considered chalky kernels. The chalky rice rate was expressed as the mean of two tests and calculated using formula (2):

$$C\left(\%\right)=\frac{C1+C2}{200}\times100\,(2)$$

where C is the chalky rice rate, and C1 and C2 are the chalky rice rates of the first and the second test, respectively.

Method for test chalkiness degree

Chalkiness degree was expressed as the percentage of the total area of a kernel. The test method for chalkiness is as follows: 10 grains of rice were randomly selected from the chalky grain, flattened on a measuring plate, and observed. The mean of the 10 grains was calculated. Chalkiness was expressed as the mean of the two tests, and calculated using formula (3):

$$C\left(\%\right)=\frac{C1+C2}{2}\times100\,(3)$$

where C is chalkiness, C1 is the mean of the first test and C2 is the mean of the second test.

Method for test gel consistency

Gel consistency is the flow length of rice gel after rice flour pasting. The test method for gel consistency is as follows: Approximately 5 g was ground into powder, passed through a 0.15-mm sieve, mixed evenly, and put into the grinding mouth preparation bottle in use. Two rice samples, each 100 mg, were weighed and put in the test tube separately, to which 0.2 ml of 0.025% bromothymol blue ($C_{27}H_{28}O_5SBr_2$) was added. The test

tube was shaken gently, to fully disperse the rice flour. Subsequently, 0.2 ml of 0.2 mol L^{-1} potassium hydroxide (KOH) was added to the tube and shaken by placing it on the vortex of the oscillator to thoroughly mix. The tube was then put into the boiling water, covering it with a glass marble, and heated for approximately 8 min, ensuring that the surface of the rice glue solution reached one-third to half of the test tube's height. The test tube was removed from the boiling water and the glass marble removed. The tube was cooled for 5 min and then placed in iced water to cool for 20 min. After removing the test tube from iced water, it was allowed to stand at room temperature for 1 h. The length of the rice gel in the test tube was measured immediately. The error of the two measurements had to be not more than 7 mm, and the gel consistency was calculated using formula (4):

$$G(mm) = \frac{G1 + G2}{2} \quad (4)$$

where G is the gel consistency, G1 is the length of the rice gel in the first test, G2 is the length of the rice gel in the second test.

Method for test amylose content

The amylose content was determined according to GB/T15683, as described in section 8.1 of GB/T15683-1995: 10 g of milled rice was crushed and passed through an 80-mm mesh sieve, mixed evenly, and put into the grinding mouth preparation bottle in use. The samples were put

into the filter with methanol for 2 h and then dispersed on a plate after another 2 h to volatilize the residual methanol.

Method for test length-to-width ratio

Ten grains of complete rice were randomly selected. The grains were placed on the measuring plate and lined up on the ruler without leaving any gaps so that there was no head-to-head or tail-to-tail overlap. Subsequently, the length was read and the mean rice length calculated. The error of two tests had to be not more than 0.5 mm. Similarly, to measure the width, the 10 grains of rice were lined up side-by-side on the measuring plate and measured with a ruler. The mean width of rice was calculated, and the error of the two tests was not more than 0.3 mm. The length-to-width ratio was calculated using formula (5):

$$C = L1/W1 \quad (5)$$

where C is the length-to-width ratio, L1 is the length of head rice, and W1 is the width of head rice.
